# Tdp2: A Means to Fixing the Ends

**DOI:** 10.1371/journal.pgen.1003370

**Published:** 2013-03-07

**Authors:** John L. Nitiss, Karin C. Nitiss

**Affiliations:** 1 University of Illinois College of Pharmacy, Rockford, Illinois, United States of America; 2 University of Illinois College of Medicine, Rockford, Illinois, United States of America; University of Washington, United States of America

Topoisomerases carry out the high-wire act of changing DNA structure through transient DNA breaks. Breaks are needed because the topology of DNA can only be changed through cutting the DNA. Topoisomerases are well suited for this tricky enterprise; they hold on to DNA covalently, shielding DNA ends from participation in unwarranted repair signaling or reactions. A downside of this mechanism is that topoisomerases can get trapped on DNA, leading to the new hazard of topoisomerase-mediated DNA damage.

Topoisomerase-mediated damage occurs in at least two important ways. The best appreciated mechanism of trapping topoisomerases on DNA is through the action of anti-cancer drugs such as etoposide [1]. These agents, termed topoisomerase poisons, lead to the accumulation of cleavage complexes, transient intermediates in the enzyme reaction cycle where the DNA is cleaved and the enzyme is covalently bound to DNA via a 5′phosphotyrosyl linkage. DNA metabolic events can also trap topoisomerases: recent work has shown that DNA damage [2], single-strand breaks [3], and mis-insertion of ribonucleotides [4] can trap topoisomerase I, while abasic sites [5] and transcription-related processes [6] may trap topoisomerase II.

The diversity of processes leading to trapping of topoisomerases suggested that cells might have specific mechanisms to repair the protein/DNA covalent complexes. An appealing mechanism is direct cleavage of the tyrosine phosphate ester bond that links topoisomerases to DNA. The first protein to have this activity, tyrosyl DNA phosphodiesterase I (Tdp1), has robust activity against 3′ phosphotyrosyl linkages generated by type 1B topoisomerases [7]. While yeast Tdp1 can also process the 5′ phosphotyrosyl–linked peptides expected to be generated by topoisomerase II or type 1A topoisomerases [8], the activity of the mammalian protein against this type of linkage remains controversial [9], [10]. Subsequent work identified a second tyrosyl DNA phosphodiesterase, Tdp2, with activity against 5′ phosphotyrosyl linkages. In in this week's issue of *PLOS Genetics*, Gómez-Herreros and colleagues show the importance of Tdp2 in the repair of topoisomerase II covalent complexes [11].

Tdp2 was identified by Cortés-Ledesma and colleagues by a genetic screen using expression of a human cDNA library in yeast followed by selection for camptothecin resistance of a yeast strain lacking *tdp1* and *rad1* [12]. In addition to Tdp1, they identified a second gene previously identified as TTRAP (TRAF and TNF receptor-associated protein) [13]. They demonstrated that TTRAP had tyrosyl DNA phosphodiesterase activity for both 3′ phosphotyrosyl– and 5′ phosphotyrosyl–linked oligonucleotides, and therefore termed the protein Tdp2. A key finding from the original identification of Tdp2 was that the protein was more active in processing 5′ phosphotyrosyl–linked oligonucleotides, and that siRNA knockdown of Tdp2 in mammalian cells resulted in sensitivity to etoposide, a drug targeting topoisomerase II, but not camptothecin, a drug that targets topoisomerase I. Recent work has greatly enhanced our understanding of the biochemistry and structural biology of Tdp2. Gene knockouts have been described in (avian) DT40 cells [14] and mouse [15], confirming etoposide sensitivity of vertebrate cells lacking Tdp2. In addition, DT40 cells lacking Tdp2 are hypersensitive to camptothecin only if they also lack Tdp1. The structures of Tdp2 from *C. elegans*, zebrafish, and mouse have been determined, indicating an active site that accommodates adducted single-strand DNA [16], [17]. Structural and biochemical studies indicate that Tdp2 nuclease activity is closely related to the AP endonuclease APE1 [17], [18]. Unlike Tdp1, Tdp2 shows no nuclease or nucleosidase activity.

**Figure 1:**
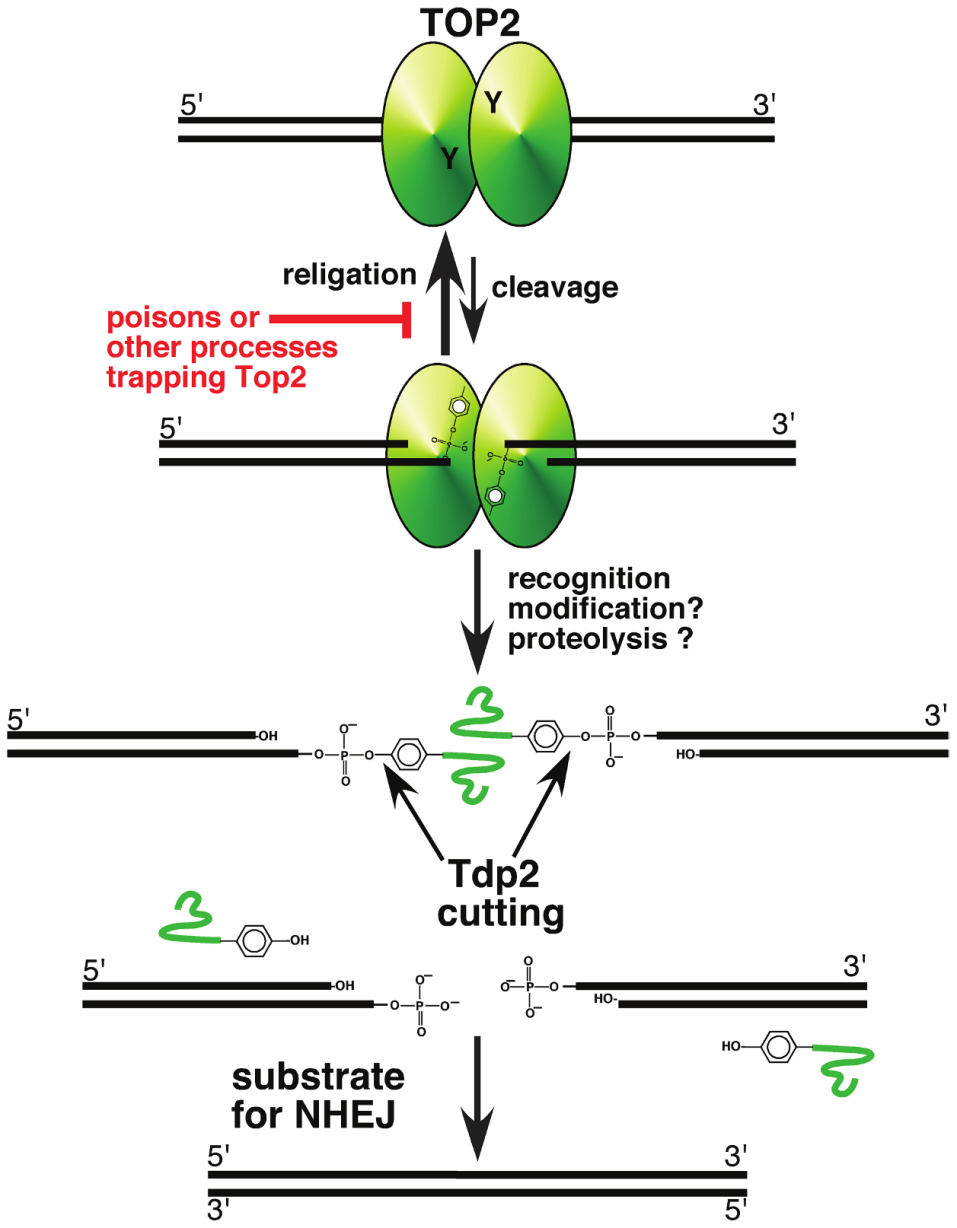
A pathway for repairing topoisomerase II–mediated DNA damage. Topoisomerase II, a homodimeric protein, cleaves both strands of DNA, and generates a four base overhang. In the absence of perturbation (such as topoisomerase poisons), religation of the broken strands is kinetically favored, and is likely inhibited by topoisomerase poisons. Recognition of the trapped protein may trigger modification and proteolysis, leaving a short peptide covalently bound to DNA. This peptide can be removed by Tdp2 cleavage of the tyrosyl phosphate bond, leaving DNA with a double-strand break. If the broken DNA is not processed prior to ligation, e.g., by DNA ligase IV in the NHEJ pathway, the result will be error-free repair of the trapped covalent complex.

Gómez-Herreros and colleagues extended the genetic analysis of Tdp2 using the knockouts in DT40 cells and mouse alluded to above. In the accompanying paper [11], the authors characterize the effects of topoisomerase II poisons such as etoposide and doxorubicin in *tdp2*-deficient cells. They confirm that *tdp2*-deficient cells are hypersensitive to topoisomerase II poisons, but not other types of DNA damage. Since removal of Top2 will result in a double-strand break (DSB) (see Figure 1), the authors postulated that Tdp2 might function in concert with a specific DSB repair pathway. Indeed, in DT40 cells, they found an epistatic relationship between Tdp2 and Ku70, a component of the non-homologous end-joining (NHEJ) pathway, in the repair of trapped Top2 covalent complexes. In other words, *tdp2 ku70* double mutants had the same sensitivity to etoposide as ku70 single mutants. If Tdp2 repaired complexes are preferentially repaired by NHEJ, then loss of Tdp2 should lead to enhanced repair of Top2 complexes by homologous recombination. This was seen, as evidenced by an increase in Rad51 foci in etoposide-treated cells lacking Tdp2 compared to wild type cells, as well as an increase in sister chromatid exchanges. Finally, Gómez-Herreros and colleagues were able to demonstrate the importance of Tdp2 in mice treated with etoposide. Tdp2-deficient mice treated with a relatively low dose of etoposide showed substantial intestinal and lymphoid toxicity compared to mice carrying wild type Tdp2. Taken together, these results clearly demonstrate the importance of Tdp2 in repairing Top2-mediated DNA damage, and suggest that Tdp2 processed damage might be preferentially channeled to NHEJ, perhaps allowing error-free repair of this damage.

While tyrosyl phosphodiesterases define an important mechanism for removing topoisomerases covalently bound to DNA, there are clearly other important mechanisms at play. Nucleolytic removal of Top2 by the MRN complex (in possible collaboration with Ctip/Sae2) has been suggested by studies in yeast and mammalian cells [19]–[21]. It might be expected that the MRN complex would preferentially channel repair products into homologous recombination pathways.

Can we infer the relative importance of the Tdp2-dependent pathway compared to other processing pathways? The results of Gómez-Herreros and colleagues clearly show the importance of Tdp2, both in terms of etoposide sensitivity and survival of the organism. However, while they show that *tdp2 ku70* double mutant cells had the same sensitivity to etoposide as *ku70* single mutants, they also find that *tdp2* single mutants were much less sensitive to etoposide than *ku70* single mutants. This implies that other pathways are important contributors to processing Top2 complexes besides Tdp2. It should also be noted that their previous work in DT40 cells seems to exclude Tdp1 as an important processing factor [15]. Taken together, these observations suggest other pathways that can process Top2 complexes that are subsequently repaired by NHEJ.

The identification of Tdp2 as a key player in repair of topoisomerase-mediated damage is also important because it provides a necessary tool for working out complete pathways. It has been suggested that an initial step in repairing topoisomerase-mediated damage is covalent modification of the trapped protein by ubiquitylation or other small ubiquitin-like proteins [22]. This recognition of topoisomerases by ubiquitin ligases is likely related to how cells avoid attempting to repair a topoisomerase that is not trapped on DNA but is undergoing a normal reaction cycle, but details of this specificity are not currently understood.

A final intriguing question concerns other functions of Tdp2. Before Tdp2 was identified as a topoisomerase repair protein, it had also been found in other contexts. Tdp2 had been identified as a protein that interacts with CD40, tumor necrosis factor (TNF) receptor-75, and TNF receptor-associated factors (and was named TTRAP) [13], and was separately found as an Ets1-interacting protein (and was named EAPII) [23]. EAPII has recently been suggested to play an important role in lung cancer development, with overexpression leading to activation of the MAPK-ERK pathway [24]. Furthermore, Tdp2 has been studied in zebrafish, where it is an essential modulator of Smad3-dependent Nodal signaling gastrulation [25]. The functions of Tdp2/TTRAP/EAPII have been reviewed recently, highlighting the important biological functions of this protein [26]. Given that there are suggestions from several studies that Tdp2/TTRAP/EAPII may play essential functions [26], the finding by Gómez-Herreros and colleagues that this gene is not essential in mouse will certainly provoke additional investigation.
